# Effect of multi-lumen perfusion line on catheter-related bacteremia in premature infants: study protocol for a cluster-randomized crossover trial

**DOI:** 10.1186/s13063-019-3218-6

**Published:** 2019-02-11

**Authors:** Aurélie Maiguy-Foinard, Bertrand Décaudin, Pierre Tourneux, Bernard Guillois, Thierry Blanc, Sophie Galène-Gromez, Morgane Masse, Pascal Odou, Fannette Denies, Benoît Dervaux, Alain Duhamel, Laurent Storme

**Affiliations:** 1 Faculty of Pharmacy, EA 7365, Groupe de Recherche sur les formes Injectables et les Technologies Associées (GRITA), University of Lille Nord de France, F-59000 Lille, France; 2 Department of Pharmacy, University Hospital Center of Lille, CHU Lille, Institut de Pharmacie, F-59000 Lille, France; 3 Department of Neonatology, CHU Lille, Jeanne de Flandre Hospital, F-59000 Lille, France; 4 EA 4489 - Environnement Périnatal et Santé, University of Lille, CHU Lille, F-59000 Lille, France; 5 Department of Neonatal Pediatrics and Intensive Care, Amiens University Hospital Center, Amiens, France; 6 Department of Neonatal Pediatrics and Intensive Care, Caen University Hospital Center, Caen, France; 7 Department of Neonatal Pediatrics and Intensive Care, Rouen University Hospital Center, Rouen, France; 8 Délégation à la Recherche Clinique et à l’Innovation (DRCI), CHU Lille, F-59000 Lille, France; 9 EA 2694 - Santé publique: épidémiologie et qualité des soins, University of Lille, CHU Lille, F-59000 Lille, France

**Keywords:** Premature infants, Catheter-related bacteremia, Infusion device, Neonatal intensive care, Central venous catheter, Drug infusion systems

## Abstract

**Background:**

Catheter-related bacteremia (CRB) is the most frequent nosocomial infection in neonatal intensive care unit (NICU) patients, especially in very low-birth-weight infants. Administration of injectable drugs in premature newborn infants has many particularities and several types of infusion incidents have been reported. The Edelvaiss® Multiline NEO device is a novel multi-lumen access infusion device adapted to the specificities of infusion in neonatology. This multicenter, randomized, controlled study was therefore designed to determine whether or not Edelvaiss® Multiline NEO reduces the risk of CRB in preterm newborn infants in an NICU.

**Methods/design:**

This is a multicenter, randomized, controlled trial, using a cluster-randomized crossover design. Four investigator centers (four clusters) will participate in the study and will be randomized into two groups, corresponding to two different sequences (either the Edelvaiss® Multiline NEO or standard infusion system sequence, then vice versa). A total of 280 patients will be recruited. Infants will be enrolled in the study at the time of placing a single-lumen central venous catheter. Three visits recording specific data are planned in the study protocol. The primary outcome measure is the incidence density (ID) of CRB. For each patient, the total number of catheters and CRB incidents as well as the duration of stay in the NICU will be computed and considered for analysis.

**Discussion:**

The study will provide high-quality evidence to determine whether the Multiline NEO device reduces the risk of CRB in preterm newborns in NICUs or not.

**Trial registration:**

ClinicalTrials.gov, NCT02633124. Registered on 7 December 2015.

**Electronic supplementary material:**

The online version of this article (10.1186/s13063-019-3218-6) contains supplementary material, which is available to authorized users.

## Background

Catheter-related bacteremia (CRB) is the most frequent nosocomial infection in neonatal intensive care unit (NICU) patients, especially for very preterm infants whose weight is very low. The incidence density (ID) of CRB is estimated at 18.5 per 1000 catheter-days for infants under 29 weeks’ gestation, according to the criteria of the monitoring Network of Néocat [1]. CRB occurs after a few days or weeks of infusion [1]. Infection is the most common serious complication of central venous catheters (CVCs).

Nosocomial CRB in NICUs contributes significantly to hospital morbidity, for example, patent ductus arteriosus, prolonged ventilation, prolonged intravascular access, bronchopulmonary dysplasia, and necrotizing enterocolitis [2]. Leroyer et al. [3] completed a study in a French NICU and estimated that infected neonates stayed on average 5.2 days more in hospital than uninfected neonates. According to this study, infections cost on average $10,440 per infected case [3, 4]. Moreover, infections (late-onset and early-onset sepsis) in very preterm infants are associated with a higher risk of adverse neurodevelopment at the age of five years [5].

Administration of injectable drugs to premature newborn infants presents numerous difficulties, for example, the need for multi-infusion intravenous (IV) therapies and very low infusion rates, limited vascular access sites, and the need to secure the immediate environment around the newborn (elevated temperature and humidity, phototherapy). Catheter manipulations are identified as an essential risk factor for CRB. To this end, Mahieu et al. [6] conducted a study in an NICU on the relationship between infusion line manipulation and CRB. The number of manipulations per catheter ranged from 30.7 up to 300, varying between 0 and 15 per day during the catheterization period. This number of manipulations was significantly different between patients with and without CRB (70.7 vs 28.7, *p* < 0.001) and increased significantly with decreased birth weight. The authors of this work show that some manipulations as well as CVC disconnections increase the risk of CRB. Several types of infusion incidents have been reported, such as hemodynamic instability in infants receiving catecholamines, excessive blood-glucose fluctuations associated with insulin infusion, CRB, and occlusion of the infusion line, to mention a few [7, 8]. According to Kalikstad et al. [9], only 4% of coadministered drugs in NICUs demonstrate unrestricted compatibility. The co-administration of incompatible drugs can have serious consequences for the patient, as drugs may not be totally administered and such incidents require manipulation of infusion lines. Septimus et al. have described several strategies to reduce CRBs, such as minimizing superfluous line manipulations [10]. Preventing particle infusion resulting from drug incompatibilities was found to reduce morbidity and mortality and the length of stay in pediatric intensive care units [11].

**Fig. 1:**
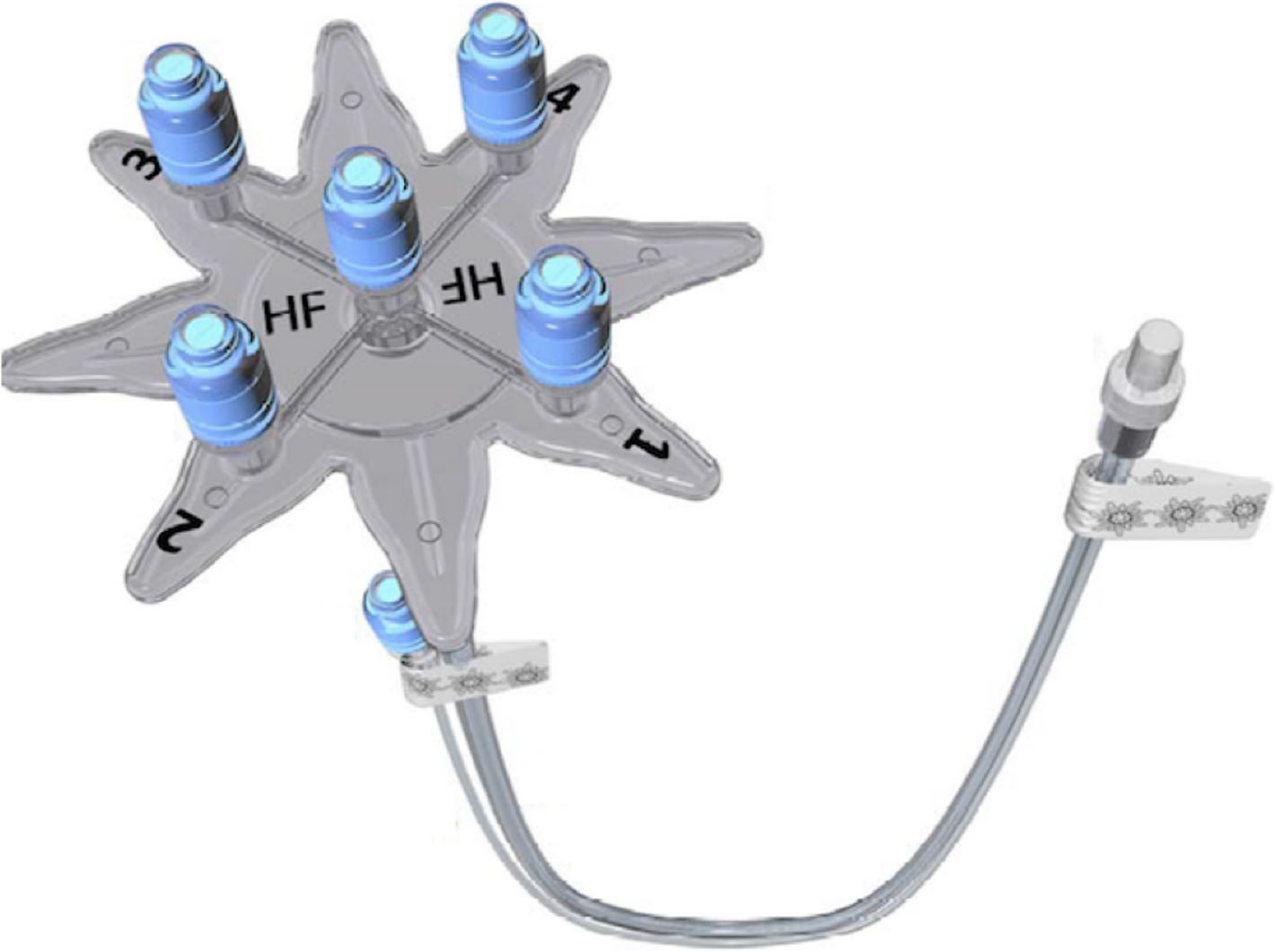
Edelvaiss® Multiline NEO (Doran International, Toussieu, France)

The Edelvaiss® Multiline NEO (Doran International, Toussieu, France) is a novel multi-lumen access infusion device adapted to the specificity of infusion requirements in neonatology (Fig. 1). This device is a follow-on to the Edelvaiss® Multiline-8 device (Doran International, Toussieu, France). Its design has been validated by the multidisciplinary working groups of the four investigation centers as far as the requirements of an NICU are concerned. It has five ports connected to five separate lumens enclosed in a single tube of 90 cm, as well as a small single tube called the annex port. Four ports (numbered 1 to 4) are connected to four peripheral lumens (residual volume per lumen 0.6 mL). The fifth port, called the central access indicated by the HF (high flow) icon, is to administer parenteral nutrition and is connected to the central lumen (residual volume 4.5 mL). The annex port enables administration to be nearer to the infant (residual volume 0.40 mL) and is intended for emergencies and direct intravenous injections. The Multiline NEO is directly connected to all single lumen CVCs through a luer lock connection.

The use of the Multiline NEO device should reduce the rate of CRB for the following reasons. First, it will require less handling of the IV administration lines. Indeed, several studies have already demonstrated that Edelvaiss® Multiline-8 can prevent variations in drug mass flow rate [12] and the occurrence of drug incompatibilities in multi-infusion IV therapy [13, 14]. The Multiline NEO device would reduce the number of perfusion interventions (such as connection/disconnection of perfused drugs) and thus the frequency of infusion line manipulations within the incubator, a direct source of bacteremia incidents. Second, the access points of the Multiline NEO device are outside the incubator at a distance from the device/catheter connection and at room temperature and relative humidity, which decreases the risk of contamination, as incubator temperature favors bacteria proliferation. Third, the lifetime of the Multiline NEO device is validated for a 21-day period of use according to the manufacturer’s indications, which reduces connections/disconnections on the CVC. These characteristics, consistent with literature data, support the hypothesis of a reduction in CRB rate.

The aim is therefore to study the efficacy of Edelvaiss® Multiline NEO over standard infusion devices in reducing the risk of CRB in preterm newborn infants < 28 ± 6 weeks admitted to an NICU. The trial was registered with the identifier NCT02633124 in ClinicalTrials.gov.

## Methods/design

### Study design

**Fig. 2:**
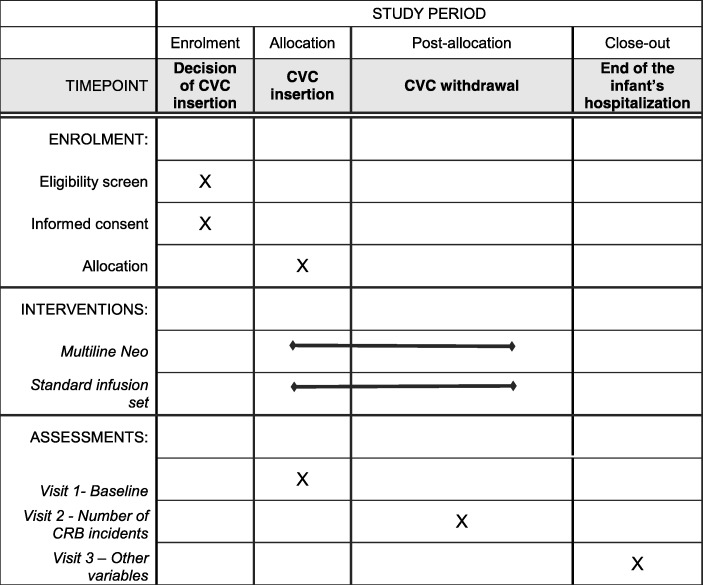
Schedule of enrolment, interventions, and assessments

The Multiline NEO study is a multicenter, cluster-randomized cross-over trial (Multiline NEO versus standard infusion system) over 28 months (Fig. 2). Four investigation centers (four clusters) will participate in the study and will be randomized into two groups to implement two different sequences. The randomization sequence will be performed using a computer-generated table provided by an independent statistician. The first group of two centers will apply the following sequence: Edelvaiss® Multiline NEO in the first period and standard infusion system in the second period. The second group of the other two centers will apply the inverse sequence: standard infusion system in the first period and Edelvaiss® Multiline NEO in the second. Each center will recruit the same number of subjects in period 1 and period 2, to obtain the same number of subjects per infusion system in each center.

The infusion system (standard infusion system and Edelvaiss® Multiline NEO) that has been assigned randomly will be used until the removal of the CVC. If a CVC is changed during a multiline sequence, another multiline NEO and a standard infusion system will also be changed. The sequence will be changed after the last patient has been enrolled in the first sequence. The primary outcome is assessed from insertion of the CVC until its withdrawal. Contamination bias between the two periods is not expected as the conditions of use (connection and disconnection of infusion lines, flushing, decontamination of access ports, etc.) are similar for each group, except for two differences: 1) access to the infusion lines is outside the incubator with the Edelvaiss® Multiline NEO, whereas they are inside with the standard infusion line; and 2) the approved lifetime (CA marking) is 4 days for the standard infusion system and 21 days for the Multiline NEO.

Infants will be enrolled in the study at the time of placing a single-lumen CVC. The catheter is connected to either the Multiline NEO device or the standard infusion system. The single-lumen catheter is typically placed between the third and sixth day of life.

The trial was registered in ClinicalTrials.gov prior to recruitment, and trial reporting will be guided by the CONSORT Statement [15]. Protocol version 1, dated April 22, 2015, was approved by the Committee for the Protection of Persons (CPP) Nord Ouest IV (CPP no. 15/29) and French National Agency for Medicine and Health Product Safety (ANSM; ID-RCB number 2015-A00585–44). Modified protocol version 2, dated September 15, 2015, was approved by the CPP Nord Ouest IV on October 13, 2015 and ANSM on October 19, 2015. Modified protocol version 3, dated May 10, 2016, was approved by the CPP Nord Ouest IV on June 14, 2016.

Informed written consent is required from both parents for enrolment in the study.

### Participant eligibility

To be fully eligible for participation in the trial, patients must meet all the following inclusion criteria and none of the exclusion criteria.

#### Inclusion criteria

1. Infants with gestational ages between 24^+ 0^ and 28^+ 6^ weeks.
2. Infants requiring a CVC in the near future. The single lumen catheter is usually placed between the third and sixth day of life
3. Informed written parental consent.

#### Exclusion criteria

1. Infants with a multi-lumen central venous catheter.
2. Infants with an umbilical venous catheter.
3. Infants with two central venous catheters.
4. No written consent from parents.

### Recruitment

Recruitment to the trial started in January 2016 and was estimated to end in December 2017 but is still ongoing. Infants will be screened for eligibility according to the inclusion and exclusion criteria. Parents will be thoroughly informed of the details of the study and its potential benefits and risks. Only infants who meet the inclusion criteria and whose parents have voluntarily provided informed written consent will be included. This study will be conducted in the NICUs of four university hospitals (Lille, Caen, Rouen, and Amiens).

### Sample size estimation

To meet the main objective, we will compare the incidence density (ID) of CRB in the two groups (Multiline NEO and standard infusion system). Determination of the initial risk level is based on data for patients under 29 weeks’ gestational age following the criteria of the Néocat network. With the standard infusion system, the CRB rate is estimated at 18.5 per 1000 catheter-days for this population. It is estimated that the Multiline NEO, correctly implemented, should reduce this rate by 50%. The average duration of catheterization is estimated at 21 days in very low-birth-weight infants. As regards the 50% reduction in CRB rate, there are even more optimistic results to be found in the literature with strategies based on structured methods to improve the care process (bundles and checklists). Erdai et al. [16] showed a 77% reduction in the rate of catheter-related infection with the use of such strategies.

As a result, with a combination of the Edelvaiss® Multiline NEO device and a training program before use, we can assert that the assumption of a 50% decrease in CRB is realistic.

Using a chi-square test at 5% significance level, with 80% power, 2510 catheter-days are required for each group. Given an average duration of 21 days of catheterization, it is therefore necessary to recruit 120 subjects per group (per drug infusion system) without clustering correction. To include clustering effects, we will use the method proposed by Giraudeau et al. [17], estimating the intra-class correlation coefficient (ICC) at 0.01 based on a previous cluster-randomized study in an ICU [18] and the inter-period correlation coefficient at 0.005 (half of ICC as recommended by Giraudeau et al. [17]). This yields 35 as the required number of subjects for each ICU and each period (design effect of 1.16). We therefore plan to recruit a total of 280 patients (140 per group).

### Study procedure

The infants’ healthcare providers will decide the date of insertion of the CVC according to NICU protocol. The choice of standard infusion system and Multiline NEO will be made randomly and will be connected to the CVC. The investigators will ensure daily that randomization is respected. The infusion system (standard or Multiline NEO) will be changed according to the manufacturer’s instructions throughout the patient inclusion period. The duration of use of the device is from the time of CVC insertion until catheter removal. Each intervention on the line (drug infusion, infusion rate, etc.), the catheter, and its dressing are recorded prospectively throughout the study period.

Three visits are planned in the study protocol:

- Visit 1 at the time of CVC insertion and onset of infusion (standard or Multiline NEO).
- Visit 2: at the withdrawal of CVC and infusion system (standard or Multiline NEO). The date and time of removal of the device, justification for CVC withdrawal (catheter removal, end of hospitalization, etc.), number of CRB incidents within the period of use of the device, number of occlusions of infusion devices and number of infusion days, and number of septic shock incidents defined by the use of vasoactive drugs will be recorded.
- Visit 3: at the end of the infant’s hospitalization. The total duration of oxygen therapy, mechanical ventilation, and parenteral nutrition, rate of chronic lung diseases, and the total length of hospital stay (including hospitalization outside the NICU) will be recorded.

### Outcome measures

#### Primary outcome

The primary outcome measure is the incidence density (ID) of CRB. CRB will be defined according to the French Néocat Network [1], whose criteria are:

- Case 1: combination of bacteremia (irrespective of blood culture site) and a positive culture from the insertion site of the CVC or umbilical catheter to the same bacterium.
- Case 2: combination of bacteremia (irrespective of blood culture site) and a positive culture (≥ 10^3^ UFC/mL in the Brun-Buisson quantitative method or ≥ 15 UFC in the semi-process -quantitative of Maki) from the CVC to the same bacterium (upon withdrawal).
- Case 3: ratio of a combination of bacteria and quantitative central blood culture/quantitative peripheral blood culture of ≥ 5.
- Case 4: combination of bacteremia and a differential delay in the positivity of the central and peripheral blood cultures ≥ 2 h.
- Case 5: absence of criteria 1 to 4 and isolation of any microorganism in at least one blood culture, with clinical and/or biological signs and establishing of suitable antibiotherapy for at least 5 days. This is the most common case.

#### Secondary outcomes

1. Number of occlusions of the infusion system defined by an acute increase in infusion pressure recorded by the pump not due to accidental kinking of the line or CVC and requiring a specific intervention by the nurse (line-flushing or change of the line or CVC).
2. Number of septic shock events defined by the occurrence of shock (low blood pressure, tachycardia, decrease in diuresis, capillary refill time > 3 s) in a context of sepsis, and the need for vasoactive drugs.
3. Total duration of oxygen therapy calculated from inclusion of the patient in the study until the end of hospitalization. Need for oxygen therapy is defined by O_2_ administration to target arterial oxygen saturation between 90 and 96%.
4. Total duration of intubation and mechanical ventilation determined from inclusion of the patient in the study until the end of hospitalization.
5. Total duration of parenteral nutrition determined from inclusion of the patient in the study until the end of hospitalization. Parenteral nutrition is stopped (and CVC retrieved) when enteral feeding is above 120 ml/kg and caloric intake is above 100 kcal/kg.
6. Presence or absence of chronic lung disease determined from inclusion of the patient in the study until the end of hospitalization. Chronic lung disease is defined by a need for O_2_ supplementation to maintain SpO2 > 90% at a postconceptional age > 36 weeks.
7. Direct costs, including components of the infusion lines (considering their replacement in case of CRB), nursing time, biological tests to identify CRB (blood culture, catheter culture), management of bacteremia, extra-time in the ICU, and increased overall length of stay in the case of CRB. Costs will be accounted for during child hospitalization. Indirect costs (e.g., presence/absence of parents) and costs induced by child handicap following a CRB are beyond the scope of the study.

### Safety and adverse events

Adverse events (AEs) are undesirable effects that happen to participants during the trial, whether or not they are considered to be related to the infusion system used (standard or Multiline NEO). All AEs during the study must be recorded on the CRF, including the nature of each event, time and date of onset, duration, intensity, criteria of gravity, assessment of cause, need for specific therapy, actions taken, and outcome. According to the severity of AEs, the investigator will determine whether the participant should be withdrawn from the study and which follow-up procedures should be performed. Again these will be recorded in detail. The relationship between the infusion system used and AEs should be assessed and recorded by the investigator.

A serious AE (SAE) is defined as any untoward occurrence or effect that causes death, is life-threatening, requires prolonged hospitalization, results in persistent significant disability, or leads to a congenital anomaly or birth defect. If an SAE occurs, the investigator must immediately report it to the principal investigator and the ethics committee and record it on the CRF with signature and date. A classification of the severity of AEs and their relationship to the infusion system under study is to be established.

An independent supervisory committee will be created. The committee will include a pediatric reanimator, a methodologist, and a pharmacist specialized in medical device vigilance.

### Data collection and management

All data will be recorded by trained clinical investigators in a standardized CRF. To ensure the accuracy and reliability of the data, the study monitor will verify and cross-check the CRFs against the investigator’s source document records. In case of any discrepancies in the cross-checking procedure, the results will be sent to the investigator for resolution. Any individual identification of the subjects will not be released until the database is closed.

Costs related to infusion (medical devices, nursing time) will be estimated by direct observation of micro-costing. Medical devices will be priced according to the invoices paid by the hospitals participating in the study. Actual wages will be taken into account to monetize nursing time. Hospital costs will be estimated from the French DRG database (ENCC). Average costs will be adjusted according to the time spent in NICUs.

### Statistical analysis

Data will be analyzed using SAS software (SAS Institute Inc., Cary, NC, USA) and all statistical tests will be performed with a two-tailed alpha risk factor of 0.05. Baseline characteristics will be described for each group. Quantitative variables will be expressed as mean (standard deviation), median (interquartile range), and range. Qualitative variables will be expressed as frequencies and percentages. Normality of distribution will be assessed graphically and using the Shapiro-Wilk test.

#### Primary outcome

The ID of CRB, expressed as the number of cases of CRB per catheter × days, will be estimated and compared for each of the two devices. The generalized linear mixed model (GLMM) will be used with the number of CRB incidents as dependent variable. The GLMM model will be parametrized with a log linear link function, a Poisson distribution, and the logarithm of catheter duration as the offset variable. To take into account the cluster crossover design, we will consider the device (standard infusion system or Edelvaiss® Multiline NEO), the period, and the ICU as fixed effects and the ICU × interaction period as a random effect as recommended by Turner et al. [19]. Effect size will be estimated by relative risk reduction with a 95% confidence interval.

#### Secondary outcomes

1. The ID of occlusion will be analyzed by the same method as for the primary objective.
2. The number of cases of septic shock and bronchopulmonary dysplasia will be analyzed using GLMM, taking into account the duration of stay in the ICU for each patient. The model will be identical to that used for the primary objective.
3. The duration of oxygen therapy, of mechanical ventilation, and of parenteral nutrition will be analyzed using the linear mixed model. The ICU, the period, and the device will be considered as fixed effects and the ICU × interaction period as a random effect. In the case of non-normal model residuals, we will use a non-parametric test without taking the design into account (Mann-Whitney U test).

Cost data will be analyzed using Stata®. Depending on normality test results, mean costs will be compared across groups using Student’s *t*-test or non-parametric bootstrap. A GLM model and a Generalized Gamma model will be used with child characteristics at baseline as regressors and considering fixed effects for the device (standard infusion system or Edelvaiss® Multiline NEO) and the ICU. Cost-effectiveness will be established and ICER estimated, retaining the number of CRB incidents as health outcomes. The 95% confidence interval for ICER will be computed using Fieller’s method or the bootstrap method.

### Ethical considerations

Informed consent will be obtained from the parent(s)/legal representative for all participants, in writing, before inclusion in the trial. Ethical approval has been obtained for the four centers from the Committee for the Protection of Persons (CPP) Nord Ouest IV (CPP no. 15/29).

## Discussion

It is essential to assess this new multi-lumen device specifically developed and designed for neonates so as to determine its effectiveness in preventing CRB in NICU patients.

Previous studies have reported the major impact of catheter manipulations on the occurrence of CRB in neonatology. In preterm and term newborn infants admitted to NICUs, the number of catheter manipulations was significantly different between patients contracting CRB or not (70.7 vs 28.7; *p* < 0.001) [6]. Moreover, CRB incidence increased significantly with decreased birth weight and gestational age. This study shows that specific manipulations (e.g., blood sampling through the central line and disconnection of the CVC) further increase the risk of CRB in neonates. Thus, the decrease in the number of infusion system manipulations will be correlated with a decrease in CRB [6].

Our hypothesis of a 50% reduction in CRB rates may not at first seem feasible but previous studies have shown an even more impressive decrease with strategies based on standardized nursing care, including bundles and checklists. As mentioned earlier, a recent study found a 77% reduction in CRB rates by implementing evidence-based measures for catheter care in an NICU [16].

The cluster-randomized crossover design of the study has several advantages. As the intervention is applied to all participants in a unit, contamination between interventions can be avoided and investigator cooperation is improved. As the interventions are applied (successively) in the same unit, a smaller number of units is required. The cluster-randomized crossover design ensures good team training in the handling of devices as their use is repeated during each period.

This trial has limitations. First, identification of CRB must be established on similar criteria. In the present study, it will be based on Neocat Network guidelines that differentiate between various types of bacteremia according to the number of cases of bacteremia and clinical or biological symptoms [20]. As special care must be taken to prevent contamination of blood culture during blood sampling, healthcare providers will have to be trained to comply strictly to the written procedure of evidence-based guidelines on blood culture sampling (Additional file 1).

## Trial status

The recruiting of patients began in January 2016 and is proceeding.

## Additional file

Additional file 1: SPIRIT 2013 checklist: Recommended items to address in a clinical trial protocol and related documents. (DOC 122 kb)

## Abbreviations

AE: Adverse event
ANSM: French National Agency for Medicine and Health Product Safety
CPP: Committee for the Protection of Persons
CRB: Catheter-related bacteremia
CRF: Case report form
CVC: Central venous catheter
DRG: Diagnosis-related groups
ENCC: French Diagnosis-Related-Groups database
GLMM: Generalized linear mixed model
ICC: Intra-class correlation coefficient
ICU: Intensive care unit
ID: Incidence density
IV: Intravenous
NICU: Neonatal intensive care unit
SAE: Serious adverse event
